# Membrane-anchored CCL20 augments HIV Env-specific mucosal immune responses

**DOI:** 10.1186/s12985-017-0831-4

**Published:** 2017-08-23

**Authors:** Xianliang Sun, Han Zhang, Shuiling Xu, Lili Shi, Jingjian Dong, Dandan Gao, Yan Chen, Hao Feng

**Affiliations:** 1 Medical school of Jiaxing University, Jiahang road 118#, Nanhu District, Jiaxing City, Zhejiang Province 314000 China; 2 Department of Microbiology & Immunology, Emory University School of Medicine, Atlanta, GA 30322 USA; 3 Jiaxing Maternity and Child Health Care Hospital, Jiaxing, Zhejiang, 314000 China

**Keywords:** Chemokine, CCL20, Antibody-secreting cells (ASCs), Mucosal immune responses, Virus-like particles (VLPs), HIV vaccine

## Abstract

**Background:**

Induction of broad immune responses at mucosal site remains a primary goal for most vaccines against mucosal pathogens. Abundance of evidence indicates that the co-delivery of mucosal adjuvants, including cytokines, is necessary to induce effective mucosal immunity. In the present study, we set out to evaluate the role of a chemokine, CCL20, as an effective mucosal adjuvant for HIV vaccine.

**Methods:**

To evaluate the role of CCL20 as a potent adjuvant for HIV vaccine, we examined its effects on antigen-specific antibody responses, level of antibody-secreting cells, cytokine production and intestinal homing of plasma cells in vaccine immunized mice.

**Results:**

CCL20-incorporated VLP administered by mucosal route (intranasal (*n* = 10, *p* = 0.0085) or intravaginal (*n* = 10, *p* = 0.0091)) showed much higher potency in inducing Env-specific IgA antibody response than those administered by intramuscular route (*n* = 10). For intranasal administration, the HIV Env-specific IFN-γ(751 pg/ml), IL-4 (566 pg/ml), IL-5 (811 pg/ml) production and IgA-secreting plasma cells (62 IgA-secreting plasma cells/10^6^ cells) in mucosal lamina propria were significantly augmented in CCL20-incorporated VLP immunized mice as compared to those immunized with Env only VLPs (*p* = 0.0332, 0.0398, 0.033, 0.0302 for IFN-γ, IL-4, IL-5, and IgA-secreting plasma cells, respectively).

Further, anti-CCL20 mAb partially suppressed homing of Env-specific IgA ASCs into small intestine in mice immunized with CCL20-incorporated VLP by intranasal (62 decreased to 16 IgA- secreting plasma cells/10^6^ cells, *p* = 0.0341) or intravaginal (52 decreased to 13 IgA- secreting plasma cells/10^6^ cells, *p* = 0.0332) routes.

**Conclusion:**

Our data indicated that the VLP-incorporated CCL20 can enhance HIV Env-specific immune responses in mice, especially those occurring in the mucosal sites. We also found that i.m. prime followed by mucosal boost is critical and required for CCL20 to exert its full function as an effective mucosal adjuvant. Therefore, co-incorporation of CCL20 into Env VLPs when combined with mucosal administration could represent a novel and promising HIV vaccine candidate.

## Background

There is currently no approved vaccine against HIV despite considerable efforts in vaccine research and clinical trial. Given the important role of mucosal surface as the port of entry for HIV, effective immunization strategies should be those that elicit significant immune responses at mucosal sites. While intramuscular(i.m) injection has been proven useful for stimulating systemic immune responses, it induces poor responses at mucosal surfaces [1]. Vaccine delivery via mucosal route provides potential opportunities for induction of effective mucosal responses. When administered in combination with systemic vaccination, mucosal immunization, including oral [2], intranasal (i.n.) [3, 4] as well as intravaginal (IV) routes [5, 6], has been proven highly potent in inducing effective mucosal immune responses.

Intensive studies have been conducted to develop and investigate potent mucosal adjuvants for improved vaccine potency. Several stimulatory molecules are suggested as effective adjuvants to enhance mucosal immune response, these including cholera toxin, *Escherichia coli* heat-labile toxin, CpG motif, and cytokines [7–9]. However, to date no licensed immunopotentiating mucosal adjuvant is available. Chemokines are a group of small proteins that play important roles in innate and acquired immunity by regulating inflammation, leukocyte trafficking, and immune cell differentiation [10–12]. Chemokine (C-C motif) Ligand 20 (CCL20), also known as Macrophage Inflammatory Protein (MIP)-3-alpha or Liver Activation Regulated Chemokine (LARC), has been shown to be expressed constitutively in variety of normal human mucosa-associated tissues, especially in the mucosal epithelial cells. CCL20 plays an important role in mucosal homeostasis through attraction of immune cells including DC, T and B-lymphocytes [13, 14]. Meanwhile, CCL20 itself is regulated. For instance, the presence of inflammatory mediators promotes the up-regulation of CCL20 in mucosal epithelial cells [15, 16]; other studies have also demonstrated that the colonic epithelial cells from patients with inflammatory bowel disease produce higher level of CCL20 [17, 18].

Neutralization of CCL20 expression by its monoclonal antibody has been shown to reduce T cell recruitment [19]. This finding demonstrates that CCL20 contributes to the enhanced recruitment of its potential target cells, which are known to home preferentially to these sites of continuous antigen-challenge, such as the epithelial mucosal surface [20]. Given the important physiological roles of CCL20 in immune regulation, leukocyte trafficking and immune cell differentiation, we set out to find out whether such biological functions could be employed to enhance mucosal immune response, especially these against HIV immunogen. In this study, CCL20 has been incorporated into HIV Env Virus-Like Particles (VLPs) and its immunomodulatory role was investigated.

## Methods

### Generation of rBVs expressing membrane-anchored CCL20 and Env

Con-s Env used in this vaccine strategy is a derivative of Con-s gp145CFI and an engineered HIV-1 group M consensus envelope gene with shortened consensus variable loops, designed to induce broad and cross-reactive HIV envelope immunity [21]. To produce the membrane-anchored CCL20, glycosylphosphatidyl-inositol-GPI domain were fused with CCL20-encoding gene in frame by overlapping PCR [22]. In addition, the coding DNA sequences of mellitin signal peptide were added to the N terminal of CCL20 and Env. The *ccl20, env or gag* gene was cloned into pFastbac1 and tranformed into *Escherichia coli* DH10 competent cells to generate the recombinant bacmid containing individual target genes, then used to transfect sf9 cells to produce recombinant baculovirus expressing protein CCL20, Env or Gag. Recombinant Baculovirus (rBVs) expressing CCL20, Env or Gag were generated using Bac-to-Bac expression system (Invitrogen, Carlsbad, USA) following the manufacturer’s protocol.

### Production and characterization of HIV VLPs

Four different HIV VLPs including Gag VLPs (control as baseline Env - associated immunity in the absence of Env or Env-CCL20), Gag/Env VLPs (standard VLPs), Gag/Env/CCL20 (chimeric VLPs, cVLPs), Gag/Env-CCL20 (standard VLP mixed with soluble CCL20) were produced by insect cell (sf9) expression system. For cVLPs, sf9 cells were co-infected with three rBVs expressing HIV Env, GPI-CCL20, and Gag at the MOI of 3:1:1. Standard VLPs were produced by co-infection of sf9 cells with rBVs expressing Env and Gag. Gag VLPs were produced by infection of sf9 cells with rBVs expressing Gag. After 48 h infection, the culture supernatant was collected and VLPs were concentrated by porous fiber filtration using the Quixstand benchtop system (GE Healthcare, Uppsala, Sweden) followed by sucrose density gradient ultracentrifugation as described previously [22]. The protein composition of VLPs was characterized by western blotting using antibodies against Gag, Env and CCL20 (R&D system), respectively. VLP protein concentration was determined by ELISA in which purified proteins were used to generate the quantitative standard curve. Bio-Rad protein assay (Bio-Rad laboratories, Inc., Hercules, USA) was used to quantify the yield of total protein in VLP.

### Immunization of mice and sampling

Female BALB/c mice (6–8 wk. old) were purchased from Beijing HFK Biotechnology (Beijing, China) and separated into three groups according to different vaccine administration routes. Group 1, mice were immunized by one i.m prime followed by two i.m boosts with HIV VLPs at intervals of 4 weeks. Group 2 or 3 mice were immunized by a single i.m prime followed respectively by two i.n or IV boosts with HIV VLPs at intervals of 4 weeks. Within each of these groups, mice were further divided into four subgroups (10 mice/subgroup) and immunized by different HIV VLPs (Table 1). On average, mice in the Gag only VLP immunized group were immunized with 100 μg of total protein. Standard and chimeric VLPs were administered by doses containing 10 μg Env. One dose of CCL20-containing VLP (chimeric VLPs) or standard VLPs with soluble CCL20 contained approximately 1.5 μg of CCL20. Two weeks after each immunization, blood samples were collected by retro-orbital plexus puncture, and sera samples were collected from the non-anticoagulated blood following a brief centrifugation at 3000 rpm for 5 min. The collected sera were stored at −20 °C until further analysis. The vaginal lavages were collected by lavaging 200 μl of PBS intravaginally using oral feeding needles. Samples were briefly centrifuged and the supernatant was filtered through a 0.22 μm filter and stored at −80 °C until further analysis.

**Table 1:** Immunization Regimens and Schedule

	Group 1	Group 2	Group 3
Boost Route	i.m.	i.n.	IV
	Gag VLPs	Gag VLPs	Gag VLPs,
4 Subgroups (10 mice/subgroup) Based on Vaccine	Gag/Env VLPs Gag/Env/CCL20 VLPs Gag/Env-CCL20 VLPs	Gag/Env VLPs Gag/Env/CCL20 VLPs Gag/Env-CCL20 VLPs	Gag/Env VLPs, Gag/Env/CCL20 VLPs Gag/Env-CCL20 VLPs
Schedule	One i.m prime followed by two i.m boosts at intervals of 4 weeks	One i.m prime followed by two i.n. boosts at intervals of 4 weeks	One i.m prime followed by two IV boosts at intervals of 4 weeks
Anti-CCL20 IgG	Three hours after each immunization	Three hours after each immunization	Three hours after each immunization

The mice were separated into three groups according to administration routes. Group 1, mice were immunized by one i.m prime followed by two i.m boosts with HIV VLPs. Group 2 or 3 mice were immunized by a single i.m prime followed respectively by two i.n or IV boosts with HIV VLPs. Within each of these groups, mice were further divided into four subgroups and immunized by different HIV VLPs

To explore the role of CCL20 in intestinal homing of plasma cells, part of immunized mice in different immunization groups were intraperitoneally treated with monoclonal anti-CCL20 IgG 25 μg (R&D system) (R&D system) three hours after each immunization.

The animal experiments was approved by the Ethics Committee of Jiaxing University. The committee’s reference number was No. JUMC2016–001. All manipulation of the mice satisfied the requirements of the Regulations of Experimental Animal Administration of China.

### Measurement of Env-specific ab titers

To determine HIV Env specific IgG and IgA antibody response, ELISA assay was performed as described previously [23]. ELISA plates (NUNC MaxiSorp, Thermo scientific) were coated with Con-s Env protein (5 μg/ml, 50 μl/well) overnight. Plates were washed three times with PBS plus 0.05% Tween-20 and blocked with 1% BSA (Sigma, St Louis, USA) in PBS for 1 h at 37 °C. Following washes, serial dilutions of samples were added to the plate and incubated for 1 h at 37 °C. For IgG detection, goat HRP-conjugated anti-mouse IgG antibody (Jackson Immuno-Research, West Grove, USA) was added and incubated for 1 h at 37 °C. For IgA detection, goat HRP-conjugated anti-mouse IgA antibody (Sigma, St Louis, USA) was added and incubated for 1 h at 37 °C. Second antibody were diluted at 1:2000. After thorough wash, the tetramethylbenzidine (TMB, R&D Systems, USA) was added and the OD value was read in an ELISA plate reader using a test wavelength of 450 nm. The highest dilution factor that gives an OD 450 of twice that of the naive sample at the dilution was designated as the antibody end point titer.

### Isolation of lamina propria lymphocytes

Two weeks after the final immunization, mice were anesthetized and sacrificed. Lymphocytes were prepared from intestinal lamina propria as previously described [24]. Intestinal sections were opened longitudinally, cut into 0.5-cm pieces, and washed twice in calcium- and magnesium-free PBS. The pieces were transferred to 75-ml tissue culture flasks containing 30 ml of Hanks’ balanced salt solution (HBSS; Gibco BRL, Grand Island, USA) containing 0.75 mM anhydrous ethylenediamine tetraacetic acid (EDTA) (Sigma, St. Louis, USA), 100 U/ml penicillin, 100 mg/ml gentamicin, 25 mM HEPES buffer, and 5% fetal calf serum (FCS). Flasks were incubated at 37 °C with rapid shaking (300 rpm) for 30 min, fresh HBSS–EDTA was added to the intestinal pieces, and the process was repeated at least twice. LPL were collected by cutting the remaining intestinal segments into 1- to 2-mm pieces using paired scalpel blades, and the fragments were transferred to sterile 75-mm tissue culture flasks with RPMI 1640 medium containing 15 U/ml collagenase (type II, Sigma, St Louis, USA), penicillin, gentamicin, HEPES buffer, L-glutamine, and 5% FCS. The flasks were incubated at 37 °C for consecutive 30-min intervals. At the end of each interval, intestinal pieces were further disrupted by pumping the pieces up and down 15 times in a 10-ml pipet. The medium (containing LPL) was separated from the remaining tissue fragments, washed in RPMI-5, and stored on ice 5 min. This process was repeated three times, until the intestinal pieces had completely dissociated into small fragments. The lymphocytes were enriched by discontinuous Percoll (Sigma, St Louis, USA) density gradients, which were prepared by 2 ml of 60% Percoll (*v*/v, diluted in RPMI-1640 topped with 2 ml of 35% Percoll (*v*/v, diluted in RPMI-5) in 15-ml centrifuge tubes. The LPL cell preparations were resuspended in 10 ml of RPMI-5, layered on the Percoll gradients, and centrifuged at 800 g for 20 min at 4 °C. The interface between the 35% and 60% gradients (containing the lymphocytes) was collected by careful pipetting. Cells were then washed in 50 ml PBS, counted and resuspended in RPMI-5 at 1 × 10^7^ cells/ml on ice until use. All lymphocytes were >90% viable by trypan blue exclusion. These cells were used immediately for ELISPOT assay.

### ELISPOT

Total IgA ASCs and HIV Env-specific IgA ASCs were analyzed by ELISPOT a previously established protocol [25, 26]. Nitrocellulose 96-well plates (Multiscreen 96-well filtration Plate; Millipore, Billerica, USA) were coated with 5 μg/ml polyclonal goat anti-mouse IgA (Kirkegaard & Perry Laboratories, Gaithersburg, MD) or Env protein in PBS at 4 °C overnight. After washing and blocking with RPMI 1640 containing 10% FCS, LPL cells were suspended in RPMI 1640 and added to wells, and incubated at 37 °C overnight in humidified air with 5% CO_2_. After washing with PBS, 100 μl of biotinylated polyclonal anti-mouse IgA (Kirkegaard & Perry Laboratories) at 100 ng/ml in PBS containing 10% FCS was added to each well and incubated at room temperature for 2 h. After washing with PBS containing 10% FCS, each well was added with 100 μl of streptavidin-HRP in PBS containing 10% FCS and incubated at room temperature for 1 h. For color development after washing with PBS, peroxidase substrates were added, and the numbers of spots per well were counted under an inverted microscope.

### Cytokine assays

Env-specific IFN-γ, IL-4 and IL-5 production were measured in the supernatants from lamina propria T cells after ex vivo re-stimulation with Env using commercial ELISA kits (R&D system, Minneapolis, USA) and following manufacturer’s procedures [27].

## Results

### Characterization of HIV VLPs

**Fig. 1:**
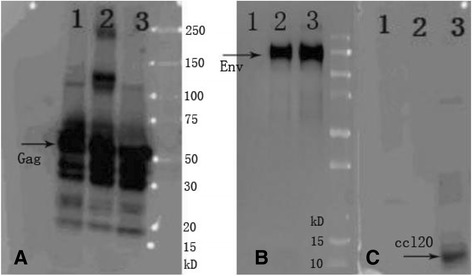
Characterization of HIV VLPs. Western blotting analysis of the protein component of HIV VLPs. The VLPs samples were loaded on SDS-PAGE followed examination by western blotting. Protein bands were probed with anti-HIV Gag antibody (panel **a**); anti-gp120 antibody (panel **b**); anti-CCL20 antibody (panel **c**); Lane 1, Gag only VLPs; lane 2, standard VLPs; lane 3, chimeric VLPs. M, molecular weight (kD). the Target bands were indicated by arrows

HIV standard and chimeric VLPs were produced by baculovirus expression system in insect cells. Gag only VLPs was observed to have a molecular mass of about 57 kDa as shown in Fig. 1a, Env (with heterologous TM/CT domains from the MMTV) incorporated into the standard VLP (Gag/Env) and chimeric VLPs (cVLPs, Gag/Env/CCL20) was observed to have a molecular mass of about 120 kDa as shown in Fig. 1b, indicated by arrow. The membrane anchored CCL20 is predicted to have a molecular mass of about 11 kDa, which was seen in cVLPs as shown in Fig. 1c.

### System and mucosal immune response

**Fig. 2:**
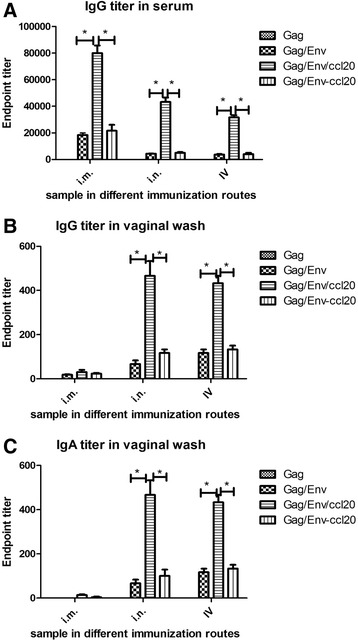
Systemic and mucosal Ab responses against HIV-gp120. The mice were immunized with Gag VLPs, standard VLPs(Gag/Env), chimeric VLPs (Gag/Env/ccl20) and standard VLPs mixed with soluble CCL20 (Gag/Env-ccl20) by different immunization routes. The sera and vaginal wash samples were collected 2 weeks after each immunization and final samples were tested in the present study. **a**, Serum IgG titers; **b** and **c**, IgG and IgA titers of vaginal wash, respectively. Assays were performed as described in materials and methods. Results are expressed as means ± standard deviations. *P* values of less than 0.05 (*p* < 0.05) were considered to be statistically significant. *, *p* < 0.05

To determine if immunization with CCL20 could enhance systemic and mucosal immune responses, serum and mucosal samples were evaluated for Env-specific IgG and IgA titers by ELISA with Env gp120 as coating antigens. As shown in Fig. 2 panels a-c, cVLPs containing Gag, Env and membrane anchored CCL20 (Gag/Env/ccl20) elicited significantly higher levels of systemic IgG and vaginal IgA/IgG responses as compared to standard VLPs (Gag/Env) without CCL20. For instance, specifically, mice boosted by i.n. with cVLPs achieved between 9 and 10-fold higher IgG titers (endpoint titers, 41,000) than mice from the Gag/Env group (endpoint titers, 4050, *p = 0.0212) and mice from Gag/Env-CCL20 group (endpoint titers, 4550, *p = 0.0265) Fig. 2a. These data also suggested that simply mixing purified CCL20 with standard VLPs (Gag/Env-ccl20) showed no significant improvement on either systematic or mucosal antibody responses. Therefore, CCL20 in its VLP-incorporated form rather than the soluble form was an effective adjuvant that enhanced both systemic and mucosal immune responses. As a note, no IgA was detected in serum samples.

The mucosal responses (Fig. 2, panels b and c) suggested the trend that i.n. and IV boost dramatically improved vaginal IgG and IgA immune responses as compared to i.m. boost. Based on the above data, we found that significantly augmented mucosal antibody responses can be achieved by mucosal boost immunization strategy in which CCL20 is integrated to Env-VLP and co-expressed as a vaccine adjuvant.

### Cytokine production by HIV VLPs stimulated lamina propria cells

**Fig. 3:**
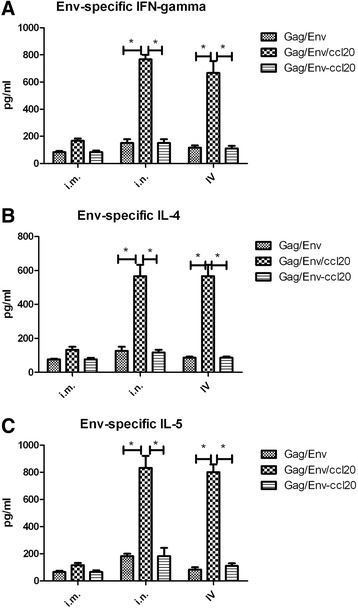
HIV gp120-specific Th1/Th2-associated cytokine production. Lamina propria cells isolated from immunized mice (1 × 10^7^cells) were stimulated with gp120 (20μg/ml) for 5 days, and cytokine production was quantified afterward. HIV gp120-specific cytokine production in HIV-VLPs treated mice is shown. Panels **a**, **b** and **c** represent the quantified IFN-γ, IL-4, IL-5 concentrations (pg/ml), respectively, in the supernatants from ex vivo HIV-VLPs re-stimulated lamina propria cells. Results are expressed as means ± standard deviations. *P* values of less than 0.05 (*p* < 0.05) were considered to be statistically significant. *, *p* < 0.05

Lymphocytes isolated from lamina propria were re-stimulated with HIV Env-gp120 to quantify Th-1 and Th-2 type cytokine production. Our results in Fig. 3 panel a showed that cVLPs, as compared to standard VLPs, significantly increased the IFN-γ production of lamina propria cells collected from i.n. and IV groups. Specifically, cVLPs immunized mice achieved 4-fold (751 pg/ml,*p = 0.0332) higher level of IFN-γ than that from the Gag/Env group (169 pg/ml). In group 1 (i.m. boost), the IFN-γ production was also enhanced in the splenic lymphocytes. Similar results were observed for IL-4 and IL-5 (Fig. 3 panels b and c). On the other hand, the standard VLP mixed with CCL20 didn’t show any significant boosting effect. Further, the mucosal boosts showed significant potency in inducing cytokine production in cVLP immunized groups. These data suggest that CCL20-incorporated HIV VLPs can effectively enhance cytokine production when administered via the intranasal or intravaginal routes of immunization.

### Env-specific IgA ASCs in lamina propria

**Fig. 4:**
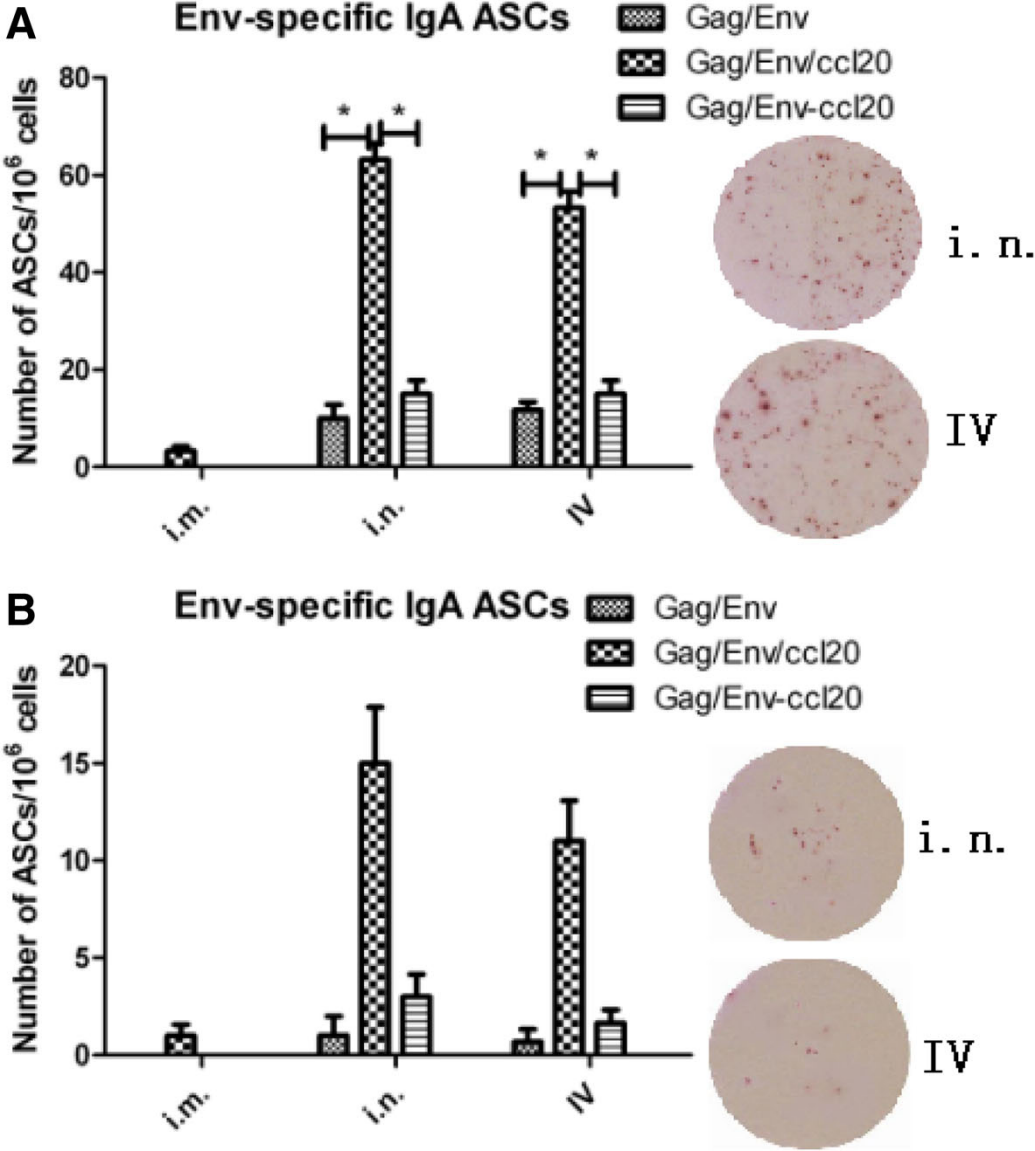
Effect of CCL20 on the level of mucosal IgA-ASCs. Distribution of Env-specific IgA ASCs at the mucosal level in BALB/c mice immunized with various HIV VLPs by different routes i.m., i.n., IV is shown in panel **a**. Lymphocytes were prepared from lamina propria of small intestine. Effect of blocking CCL20 on intestinal homing of Env-specific IgA ASCs was shown in panel **b**. BALB/c mice were immunized with HIV VLPs in different routes and also injected i.p. with PBS or anti-CCL20 mAb. After 6 days, lymphocytes were prepared from lamina propria of small intestine. IgA ASCs were enumerated by ELISPOT. The results of ELISPOT images were from cVLPs immunized groups. Data represent mean ± SD and statistically significant differences are represented

Given that antigen-specific IgA induction constitutes the main adaptive immune responses against pathogen at mucosal sites, we evaluated the distribution of Env-specific IgA ASCs induced by different immunization routes in small intestine. After the last immunization, the number of Env-specific IgA ASCs in different immunization groups was quantified by ELISPOT. As shown in Fig. 4a, both i.n. and IV immunizations by cVLPs induced increased number of Env-specific IgA ASCs suggesting that the VLP-incorporated CCL20 effectively induced chemotaxis of their responsive cells to intestine. In i.n. immunized group, incorporated CCL20-responsive Env-specific IgA ASCs showed a 4.5-fold (62 IgA-secreting plasma cells/10^6^ cells, *p = 0.0325) increase as compared to that of the mice immunized by soluble CCL20 mixed with VLPs (13 IgA-secreting plasma cells/10^6^ cells). Similar trend was seen in IV immunized mice, however, with a slightly lower number of ASCs in cVLP immunized group compared to that from i.n.-cVLP group. Our data therefore demonstrated that CCL20 may play a role in immunocyte migration to certain effector sites when administered by i.n. or IV rather than the i.m. route.

To explore the role of CCL20 in intestinal homing of plasma cells, we examined the effect of in vivo neutralization of CCL20 on homing of Env-specific IgA ASCs into intestine in mice immunized with VLPs. As shown in Fig. 4b and in comparison with the data from Fig. 4a, anti-CCL20 treated groups showed reduced numbers of Env-specific IgA ASCs, indicating that CCL20 indeed plays a role in induction of Env-specific IgA ASCs.

## Discussion

Mucosal transmission of HIV infection accounts for as high as 80% of AIDS [28]. It is generally believed that the induction of effective immune responses at mucosal portals of viral entry offers the best protection against HIV [29]. To date, an HIV vaccine with fully protective efficacy is not yet available. An effective HIV vaccine should not only induce systemic immune response to clear disseminated viruses, but also promote mucosal immune responses to block transmission. Our previous studies have shown that incorporation of cytokines as adjuvants promotes enhanced mucosal and systemic responses with improved quality [30]. The important roles that cytokines play in modulating immune responses induced by various vaccines have also been established [31]. Given the important roles that CCL20 and its receptor CCR6 play in leukocyte maturation, migration as well as mucosal immunity, we set out to investigate the potential of this chemokine as a molecular adjuvant for HIV vaccine for improved mucosal and systemic responses.

In the present study, engineered membrane-bound CCL20 was co-incorporated with HIV Env into VLPs and the resulted cVLPs were tested for their efficacy in promoting enhanced immune responses. Data from Fig. 2 strongly indicated that the co-incorporation of CCL20 into VLPs arguments both serum as well as mucosal immune responses as reflected by significantly improved IgG and IgA titers in serum as well as vaginal samples from i.n and IV boost groups. IgA is an important type of mucosal antibody involved in the first line of defense of pathogens and capable of inhibiting virus transcytosis through epithelial layers and has potent neutralizing activity [15]. Therefore, a vaccine capable of eliciting IgA response could be beneficial by contributing to the containment of HIV infection.

It is well established that T cell responses are crucial for clearing viral-infected cells and controlling viral replication. T cell responses are beneficial to host immune defense against HIV infection [32, 33], and CD4^+^ T cell responses are critical for humoral responses. We evaluated the T cell responses (Ag-specific Th1- and Th2 associated cytokine induction) induced by various VLPs. Results in Fig. 3 demonstrated that incorporated CCL20 enhanced both Th1 and Th2 responses in mice when the cVLPs were boosted by i.n. or IV routes.

To further confirm the adjuvant effect of CCL20, the level of Env-specific IgA ASCs was evaluated by ELISPOT. Our data (Fig. 4a) suggested that cVLPs induced elevated level of Env-specific IgA ASCs as compared to standard VLPs. To verify the contribution of CCL20 to the elevated level of IgA ASCs and homing of Ag-specific IgA ASCs into small intestine in mice immunized with CCL20, CCL20 was neutralized by its monoclonal antibodies. The level of Env specific IgA ASCs in the lamina propria was reduced (Fig. 4b), indicating that the homing of Env specific IgA ASCs was partially blocked by anti-CCL20 in small intestines. The impaired IgA ASCs level implied that CCL20 may play a role in improving IgA responses and the partially blockage by anti-CCL20 antibodies can be explained as the following. The chemokine and their receptors contribute to accumulation of specific leukocytes in various tissues [34], and it is well established that chemokine and tissue-specific adhesion molecules are involved in the migration of Ab-secreting cells. It is possible that some Env specific IgA ASCs are still capable of entering into intestinal tissues by responding to other chemotactic factors, such as CCL25, CCL28 and their corresponding receptors CCR9 and CCR10, which enables plasma B cells to migrate to mucosal tissues [35]. Mucosa-associated Epithelial Chemokine (MEC/CCL28), a well-known ligand for CCR10, is commonly produced by epithelial cells at several mucosal sites and attracts IgA- but not IgG or IgM-producing ASCs into the intestines, lungs, and oral cavity [36]. Others reported that CXCR4 and its ligand CXCL12 are involved in the translocation of plasma cells within the splenic and lymph node as well as in their homing to the bone marrow [12]. Additionally, down-regulation of CCR7 and CXCR5 will allow the B cells to migrate to efferent lymphatic vessels [37]. Further, CCL25 plays essential roles in intestinal homing of IgA ASCs [38]. Taken together, it is possible that the residual IgA ASCs were detectable due to other chemokine activities which can exert influence on the migration of lymphocytes.

Based on the above results, we concluded that immunization with cVLPs containing membrane anchored CCL20 induced enhanced Env-specific serum and mucosal immune responses, cytokine secretion, as well as IgA^+^ plasma cell production. It is noteworthy that, in contract to the VLP-incorporated CCL20, the soluble CCL20 has no adjuvant effect by i.n. nor IV immunization when mixed with HIV standard VLPs based on data shown in Figs. 2, 3 and 4. The different delivery approaches of CCL20 may contribute to the difference in association between CCL20 and antigen and therefore the adjuvant function of CCL20. In cVLPs, co-incorporated CCL20 is more likely to be co-presented with Env and accessed by APCs due to the established advantage of VLPs as efficient vaccine platform [39, 40]. CCL20 can bind to surface CCR6 on APC or other lymphocytes and thus effectively facilitate VLP uptake and antigen processing and presentation. In contrast, soluble CCL20 may be poorly associated with Env and may not interact with or recruit APCs as efficiently as its VLP-incorporated counterpart.

Another important question we asked in this study is whether immunization boost by i.n. or IV route is capable of inducing enhanced mucosal immune responses. The Gut Associated Lymphoid Tissue (GALT) contains the majority of T cells, and many studies have shown that GALT is the preferential target for HIV replication during infection [13, 14]. Therefore, mucosal vaccine holds great promise for effective HIV prophylactic. Our data suggest that i.n. or IV boost indeed induced highly augmented levels of IgG and IgA in vaginal wash when mice were immunized with cVLPs. In contrast, i.m. administration and boost of HIV cVLPs induced mainly systemic rather than mucosal immunity (Fig. 2). Further, i.n. or IV boost also elevated the level of Env-specific IgA ASCs in the intestine lamina propria as well as cytokine secretion (Figs. 3 and 4). It is intriguing to check out if i.n. boost, as a non-invasive route, has any different effect on mucosal immune responses compared to IV. As suggested by our data, no significant advantage of i.n. over IV boost. The primary function of the mucosal immune system is to protect the host from invading pathogens. It is well established that immunization route is critical in determining the nature of induced immune responses. Our data generally support the notion that i.m. route may favor the induction of systemic response, whereas i.n. or IV route might promote mucosal response [11].

## Conclusions

In summary, the present study demonstrated that CCL20 could be an effective mucosal adjuvant for HIV vaccine. It promotes enhanced Env-specific mucosal responses. Importantly, our data also suggest that i.m. prime followed by mucosal boost is critical and required for CCL20 to exert its full function as an effective mucosal adjuvant. In summary, co-incorporation of CCL20 into Env VLPs when combined with mucosal administration could represent a novel and promising HIV vaccine candidate.

## Abbreviations

ASCs: Antibody-secreting Cells
CCL20: Chemokine (C-C motif) Ligand 20
GALT: Gut Associated Lymphoid Tissue
LPL: Lamina propria lymphocytes
rBVs: Recombinant Baculovirus
VLPs: Virus-like Particles

## References

[1] Vaccari M, Poonam P, Franchini G (2010). Phase III HIV vaccine trial in Thailand: a step toward a protective vaccine for HIV. Expert Rev Vaccines.

[2] Schulte R, Suh YS, Sauermann U, Ochieng W, Sopper S, Kim KS, Ahn SS, Park KS, Stolte-Leeb N, Hunsmann G (2009). Mucosal prior to systemic application of recombinant adenovirus boosting is more immunogenic than systemic application twice but confers similar protection against SIV-challenge in DNA vaccine-primed macaques. Virology.

[3] Vyas SP, Gupta PN (2007). Implication of nanoparticles/microparticles in mucosal vaccine delivery. Expert Rev Vaccines.

[4] Matano T, Kobayashi M, Igarashi H, Takeda A, Nakamura H, Kano M, Sugimoto C, Mori K, Iida A, Hirata T (2004). Cytotoxic T lymphocyte-based control of simian immunodeficiency virus replication in a preclinical AIDS vaccine trial. J Exp Med.

[5] Lewis DJ, Wang Y, Huo Z, Giemza R, Babaahmady K, Rahman D, Shattock RJ, Singh M, Lehner T (2014). Effect of vaginal immunization with HIVgp140 and HSP70 on HIV-1 replication and innate and T cell adaptive immunity in women. J Virol.

[6] McKay PF, Mann JF, Pattani A, Kett V, Aldon Y, King D, Malcolm RK, Shattock RJ (2017). Intravaginal immunisation using a novel antigen-releasing ring device elicits robust vaccine antigen-specific systemic and mucosal humoral immune responses. J Control Release.

[7] Vajdy M, Singh M, Kazzaz J, Soenawan E, Ugozzoli M, Zhou F, Srivastava I, Bin Q, Barnett S, Donnelly J (2004). Mucosal and systemic anti-HIV responses in rhesus macaques following combinations of intranasal and parenteral immunizations. AIDS Res Hum Retrovir.

[8] HK TAKUTOHIKICHI, OYAMA HITOSHI, YAMAMOTO GO, WATANABE HIROSHI, IRISAWA ATSUSHI, OBARA KATSUTOSHI, SATO YUKIO (2005). EFFECTIVENESS OF INTRAGASTRIC IMMUNIZATION WITH PROTEIN AND OLIGODEOXYNUCLEOTIDES CONTAINING a CpG MOTIF FOR INDUCING a GASTROINTESTINAL MUCOSAL IMMUNE RESPONSE IN MICE. Fukushima J Med Sci.

[9] Boyaka PN, McGhee JR (2001). Cytokines as adjuvants for the induction of mucosal immunity. Adv Drug Deliv Rev.

[10] SA P. The RV144 Thai HIV vaccine trial. Hum Vaccin. 2010;6 20431337

[11] Cafaro A, Macchia I, Maggiorella MT, Titti F, Ensoli B (2009). Innovative approaches to develop prophylactic and therapeutic vaccines against HIV/AIDS. Adv Exp Med Biol.

[12] Hargreaves DC, Hyman PL, Lu TT, Ngo VN, Bidgol A, Suzuki G, Zou YR, Littman DR, Cyster JG (2001). A coordinated change in chemokine responsiveness guides plasma cell movements. J Exp Med.

[13] Dandekar S (2007). Pathogenesis of HIV in the gastrointestinal tract. Curr HIV/AIDS Rep.

[14] Centlivre M, Sala M, Wain-Hobson S, Berkhout B (2007). In HIV-1 pathogenesis the die is cast during primary infection. AIDS.

[15] Schmuth M, Neyer S, Rainer C, Grassegger A, Fritsch P, Romani N, Heufler C (2002). Expression of the C-C chemokine MIP-3 alpha/CCL20 in human epidermis with impaired permeability barrier function. Exp Dermatol.

[16] Nakayama T, Fujisawa R, Yamada H, Horikawa T, Kawasaki H, Hieshima K, Izawa D, Fujiie S, Tezuka T, Yoshie O (2001). Inducible expression of a CC chemokine liver- and activation-regulated chemokine (LARC)/macrophage inflammatory protein (MIP)-3 alpha/CCL20 by epidermal keratinocytes and its role in atopic dermatitis. Int Immunol.

[17] Izadpanah A, Dwinell MB, Eckmann L, Varki NM, Kagnoff MF (2001). Regulated MIP-3alpha/CCL20 production by human intestinal epithelium: mechanism for modulating mucosal immunity. Am J Physiol Gastrointest Liver Physiol.

[18] Kwon JH, Keates S, Bassani L, Mayer LF, Keates AC (2002). Colonic epithelial cells are a major site of macrophage inflammatory protein 3alpha (MIP-3alpha) production in normal colon and inflammatory bowel disease. Gut.

[19] Katchar K, Kelly CP, Keates S, O'Brien MJ, Keates AC (2007). MIP-3alpha neutralizing monoclonal antibody protects against TNBS-induced colonic injury and inflammation in mice. Am J Physiol Gastrointest Liver Physiol.

[20] Page G, Lebecque S, Miossec P (2002). Anatomic localization of immature and mature dendritic cells in an ectopic lymphoid organ: correlation with selective chemokine expression in rheumatoid synovium. J Immunol.

[21] Liao HX, Sutherland LL, Xia SM, Brock ME, Scearce RM, Vanleeuwen S, Alam SM, McAdams M, Weaver EA, Camacho Z (2006). A group M consensus envelope glycoprotein induces antibodies that neutralize subsets of subtype B and C HIV-1 primary viruses. Virology.

[22] Wang BZ, Quan FS, Kang SM, Bozja J, Skountzou I, Compans RW (2008). Incorporation of membrane-anchored flagellin into influenza virus-like particles enhances the breadth of immune responses. J Virol.

[23] Wang BZ, Gill HS, He C, Ou C, Wang L, Wang YC, Feng H, Zhang H, Prausnitz MR, Compans RW (2014). Microneedle delivery of an M2e-TLR5 ligand fusion protein to skin confers broadly cross-protective influenza immunity. J Control Release.

[24] Veazey RS, Rosenzweig M, Shvetz DE, Pauley DR, DeMaria M, Chalifoux LV, Johnson RP, Lackner AA (1997). Characterization of gut-associated lymphoid tissue (GALT) of normal rhesus macaques. Clin Immunol Immunopathol.

[25] Sedgwick J, Holt P (1983). A solid-phase immunoenzymatic technique for the enumeration of specific antibody-secreting cells. J. Immunol. Meth..

[26] Czerkinsky CC, Nilsson LA, Nygren H, Ouchterlony O, Tarkowski A (1983). A solid-phase enzyme-linked immunospot (ELISPOT) assay for enumeration of specific antibody-secreting cells. J Immunol Meth.

[27] Rainone V, Dubois G, Temchura V, Uberla K, Clivio A, Nebuloni M, Lauri E, Trabattoni D, Veas F, Clerici M (2011). CCL28 induces mucosal homing of HIV-1-specific IgA-secreting plasma cells in mice immunized with HIV-1 virus-like particles. PLoS One.

[28] Zagury D, Bernard J, Cheynier R, Desportes I, Leonard R, Fouchard M, Reveil B, Ittele D, Lurhuma Z, Mbayo K (1988). A group specific anamnestic immune reaction against HIV-1 induced by a candidate vaccine against AIDS. Nature.

[29] Wijesundara DK, Jackson RJ, Ramshaw IA, Ranasinghe C (2011). Human immunodeficiency virus-1 vaccine design: where do we go now?. Immunol Cell Biol.

[30] Feng H, Zhang H, Deng J, Wang L, He Y, Wang S, Seyedtabaei R, Wang Q, Liu L, Galipeau J (2015). Incorporation of a GPI-anchored engineered cytokine as a molecular adjuvant enhances the immunogenicity of HIV VLPs. Sci Rep.

[31] Zhang H, El Zowalaty ME (2016). DNA-based influenza vaccines as immunoprophylactic agents toward universality. Future Microbiol.

[32] Broxmeyer HE, Kim CH, Cooper SH, Hangoc G, Hromas R, Pelus LM (1999). Effects of CC, CXC, C, and CX3C chemokines on proliferation of myeloid progenitor cells, and insights into SDF-1-induced chemotaxis of progenitors. Ann N Y Acad Sci.

[33] Lu L, Wang LS, Cooper RJ, Liu HJ, Turner K, Weich N, Broxmeyer HE (2000). Suppressive effects of TNF-alpha, TGF-beta1, and chemokines on megakaryocytic colony formation in CD34+ cells derived from umbilical cord blood compared with mobilized peripheral blood and bone marrow. J Hematother Stem Cell Res.

[34] Vajdy M, Singh M (2006). Intranasal delivery of vaccines against HIV. Expert Opin Drug Deliv.

[35] Stevceva L, Alvarez X, Lackner AA, Tryniszewska E, Kelsall B, Nacsa J, Tartaglia J, Strober W, Franchini G (2002). Both mucosal and systemic routes of immunization with the live, attenuated NYVAC/simian immunodeficiency virus SIV(gpe) recombinant vaccine result in gag-specific CD8(+) T-cell responses in mucosal tissues of macaques. J Virol.

[36] Di Fabio S, Medaglini D, Rush CM, Corrias F, Panzini GL, Pace M, Verani P, Pozzi G, Titti F (1998). Vaginal immunization of Cynomolgus monkeys with Streptococcus Gordonii expressing HIV-1 and HPV 16 antigens. Vaccine.

[37] Wehrli N, Legler DF, Finke D, Toellner KM, Loetscher P, Baggiolini M, MacLennan IC, Acha-Orbea H (2001). Changing responsiveness to chemokines allows medullary plasmablasts to leave lymph nodes. Eur J Immunol.

[38] Hieshima K, Kawasaki Y, Hanamoto H, Nakayama T, Nagakubo D, Kanamaru A, Yoshie O (2004). CC chemokine ligands 25 and 28 play essential roles in intestinal extravasation of IgA antibody-secreting cells. J Immunol.

[39] Zhang H, Wang L, Compans RW, Wang BZ (2014). Universal influenza vaccines, a dream to be realized soon. Viruses.

[40] Li Wang HZ, Richard W. Compans and Bao-Zhong Wang. : Universal influenza vaccine- short review. J Immunol. Clin Res. 2013;1

